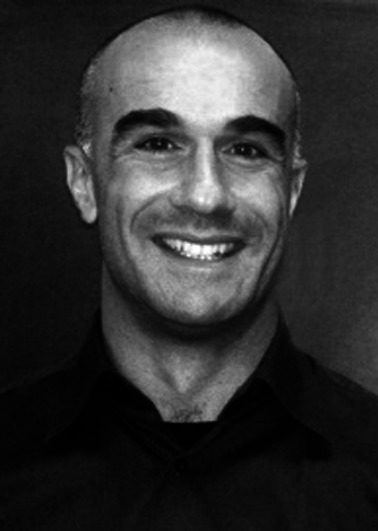# Halogen Bonding in Perovskite Solar Cells: A New Tool for Improving Solar Energy Conversion

**DOI:** 10.1002/anie.202114793

**Published:** 2022-01-19

**Authors:** Pierangelo Metrangolo, Laura Canil, Antonio Abate, Giancarlo Terraneo, Gabriella Cavallo

**Affiliations:** ^1^ Department Novel Materials and Interfaces for Photovoltaic Solar Cells Helmholtz-Zentrum Berlin für Materialen und Energie Hahn-Meitner-Platz 1 14109 Berlin Germany; ^2^ Laboratory of Supramolecular and Bio-Nanomaterials (SBNLab) Department of Chemistry, Materials, and Chemical Engineering “Giulio Natta” Politecnico di Milano Via L. Mancinelli 7 20131 Milano Italy

**Keywords:** Halogen bonding, iodine, perovskites, self-assembly, solar energy

## Abstract

Hybrid organic–inorganic halide perovskites (HOIHPs) have recently emerged as a flourishing area of research. Their easy and low‐cost production and their unique optoelectronic properties make them promising materials for many applications. In particular, HOIHPs hold great potential for next‐generation solar cells. However, their practical implementation is still hindered by their poor stability in air and moisture, which is responsible for their short lifetime. Optimizing the chemical composition of materials and exploiting non‐covalent interactions for interfacial and defects engineering, as well as defect passivation, are efficient routes towards enhancing the overall efficiency and stability of perovskite solar cells (PSCs). Due to the rich halogen chemistry of HOIHPs, exploiting halogen bonding, in particular, may pave the way towards the development of highly stable PSCs. Improved crystallization and stability, reduction of the surface trap states, and the possibility of forming ordered structures have already been preliminarily demonstrated.

## Graphical abstract

Hybrid organic–inorganic halide perovskites hold great potential for next‐generation solar cells. However, their poor stability in air and moisture still hinders their practical implementation. Preliminary data have shown that halogen bonding affords improved crystallization and stability, reduction of the surface trap states, and the possibility of forming ordered structures, paving the way towards the development of highly stable PSCs.

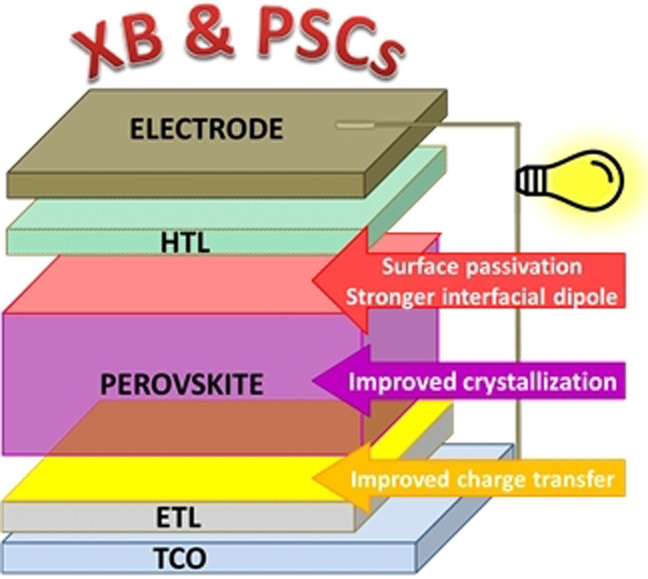

## 1 Introduction

“The fun in science lies not in discovering facts, but in discovering new ways of thinking about them.”^[1]^

All of the modern advancements in science and technology are never stand‐alone. In most cases, they have been made possible thanks to fundamental discoveries, which often are hundreds of years old. The essence of scientific progress—quoting Sir Lawrence Bragg, 1915 Nobel laureate in physics—is trying to find new ways of thinking about well‐known fundamental facts.^[1]^ In this respect, neither halogen bonding (XB) nor perovskites are new in science. They are consolidated tools in materials science and engineering, and their innovative applications opened up new possibilities for the design of advanced functional materials.[^2^, ^3^, ^4^, ^5^, ^6^]

In this Minireview we will first introduce an overview of the unique features of XB—directionality, tunability, hydrophobicity, and donor atom size—that have driven its exponential growth in the last few years. This proves the great impact of XB in several fields, opening up new opportunities for the design of supramolecular functional systems with a wide range of applications. Then, we will see how the recent combination between XB and perovskites may help overcome some of the critical issues that still prevent the practical application of perovskite solar cells. Preliminary data in this field have, in fact, already demonstrated the efficacy of XB in improving crystallization and stability, and reducing surface trap states. A bright future can therefore be foreseen for XB as a new tool for improving solar energy conversion.

## 2 The Halogen Bond

**Figure 1:**
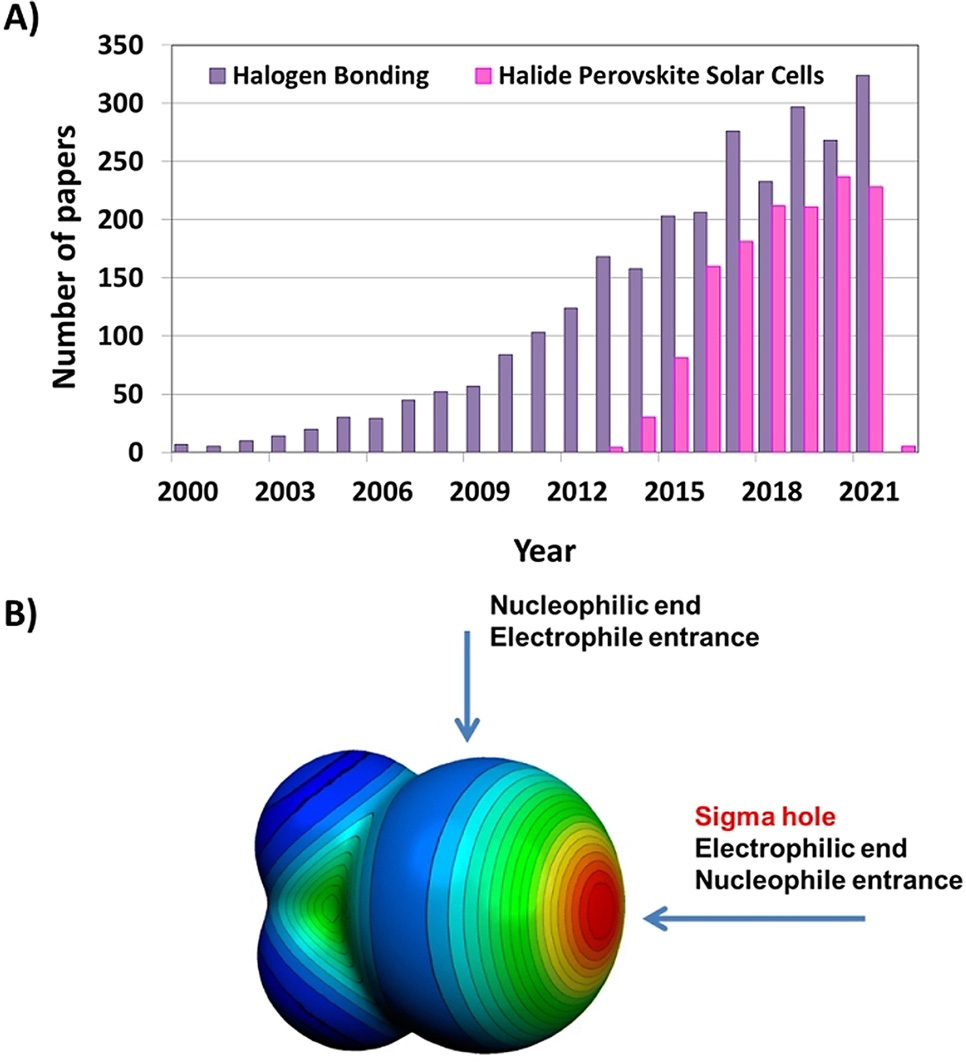
A) The incremental growth of publications having “halogen bonding” (purple) or “halide perovskite solar cells” (pink) in the title and/or abstract (source SciFinder, search performed in December 2021). B) The molecular electrostatic potential, in Hartrees, at the 0.001 electrons Bohr^−3^ isodensity surface of CF_3_I. Color ranges: red, greater than 27 kcal mol^−1^; yellow, between 20 and 14 kcal mol^−1^; green, between 12 and 6 kcal mol^−1^; blue, negative. Adapted with permission from ref. [5], https://pubs.acs.org/doi/10.1021/acs.chemrev.5b00484. Further permission related to the material excerpted should be directed to the ACS.

Within the panel of chemical interactions available to chemists, the XB, i.e., the non‐covalent interaction involving halogen atoms as electrophilic sites,^[7]^ has experienced an explosive growth in the last years. Essentially unknown before X‐ray crystallographic studies on the Br_2_⋅⋅⋅O(CH_2_CH_2_)_2_O adduct reported by O. Hassel in 1954,^[8]^ XB has quickly become a unique tool for molecular recognition processes, as shown by the increasing number of papers published per year with the topic “halogen bonding” (Figure 1 A).

A careful look at these papers shows the great evolution of XB research. At the beginning, it was just a scientific curiosity, exploited to construct beautiful supramolecular adducts.[^9^, ^10^] However, over time the attention has shifted towards the new functionalities induced in the final adducts.[^11^, ^12^] Nowadays, XB is exploited for various applications at the boundary between chemistry, nanotechnology, physics, medicinal chemistry, and molecular biology.^[5]^ This interdisciplinary character suggests that there is still much to explore about XB and research on new applications and advanced functionalities induced by XB will surely continue to grow in the next years.

The reasons for such an explosive growth lie in the unique features of XB, which cannot be easily met by other non‐covalent interactions. First of all, the anisotropic distribution of electron density in covalently bound halogen atoms allows the formation of highly directional interactions with nucleophiles (XB through the σ‐hole) and electrophiles (at the negative belt perpendicular to the covalent bond formed by the halogen) as shown in Figure 1 B.^[13]^ This high directionality, together with the tunable interaction strength enabled by the halogen atom selection, makes the XB an effective tool for controlling the self‐assembly of molecular building blocks and fine‐tuning their functional properties. Moreover, the frequent presence of fluorinated segments in the XB‐donor not only boosts interaction strength, but also increases the hydrophobicity of the final supramolecular adducts, giving protection against humidity and boosting material stability.^[12]^ Finally, the high polarizability of the heaviest halogen atoms, although it may pose some steric limitations for some applications, on the other hand, is beneficial for constructing efficient all‐organic solid‐state electronic materials. In fact, the presence of halogen atoms simultaneously allows to control the molecular packing through XB and modulate the LUMO level, lowering the HOMO–LUMO gap, thus promoting charge transport.[^14^, ^15^, ^16^, ^17^, ^18^] Further, the bare size of halogen atoms may significantly alter the light‐emitting properties of halogenated dyes by promoting singlet‐to‐triplet intersystem crossing^[19]^ and affording high phosphorescence quantum yields.^[20]^ Last, the high directionality of XB may determine the obtainment of non‐centrosymmetric structures promoting the emergence of second‐order nonlinear optical (NLO) properties.^[21]^

More recently, XB has shown its great potential in designing supramolecular photovoltaic materials with optimized morphology and charge transport and promoting efficient dye regeneration in dye‐sensitized solar cells.[^22^, ^23^, ^24^] Latest research in the field proved that XB can be successfully applied to ameliorate the performances of perovskite solar cells (PSCs), bringing innovative photovoltaic systems able to fulfil the demand for clean and sustainable energy.^[25]^

## 3 Hybrid Organic–Inorganic Halide Perovskites

Similar to XB, perovskites have been known since the 19^th^ century. However, the first evidence of their use as semiconductors for optoelectronic applications dates back only to the late 20^th^ century.[^26^, ^27^] In particular, hybrid organic–inorganic halide perovskites (HOIHPs) have emerged as promising candidates for application as lasers, light‐emitting diodes, field‐effect transistors, photodetectors, and solar cells, thanks to their easy and cheap production, high photoluminescence quantum yields, multicolor emission, and excellent excitonic and charge carrier properties (Figure 1 A).[^28^, ^29^, ^30^, ^31^, ^32^, ^33^, ^34^] In particular, HOIHPs hold great potential for next‐generation solar cells. The first application of perovskites as a light harvester in solar cells was reported in 2009,^[35]^ and since then, PSC performance has improved dramatically, reaching efficiencies higher than 25 %.^[36]^ Further enhancements of cell performances can be achieved by controlling crystal growth and surface interactions of the perovskite layer.[^37^, ^38^, ^39^] In this respect, due to the role of halide chemistry in HOIHPs, exploiting the unique features of XB in this field may be particularly useful.

### 3.1 XB Passivation of Halide Perovskites

**Figure 2:**
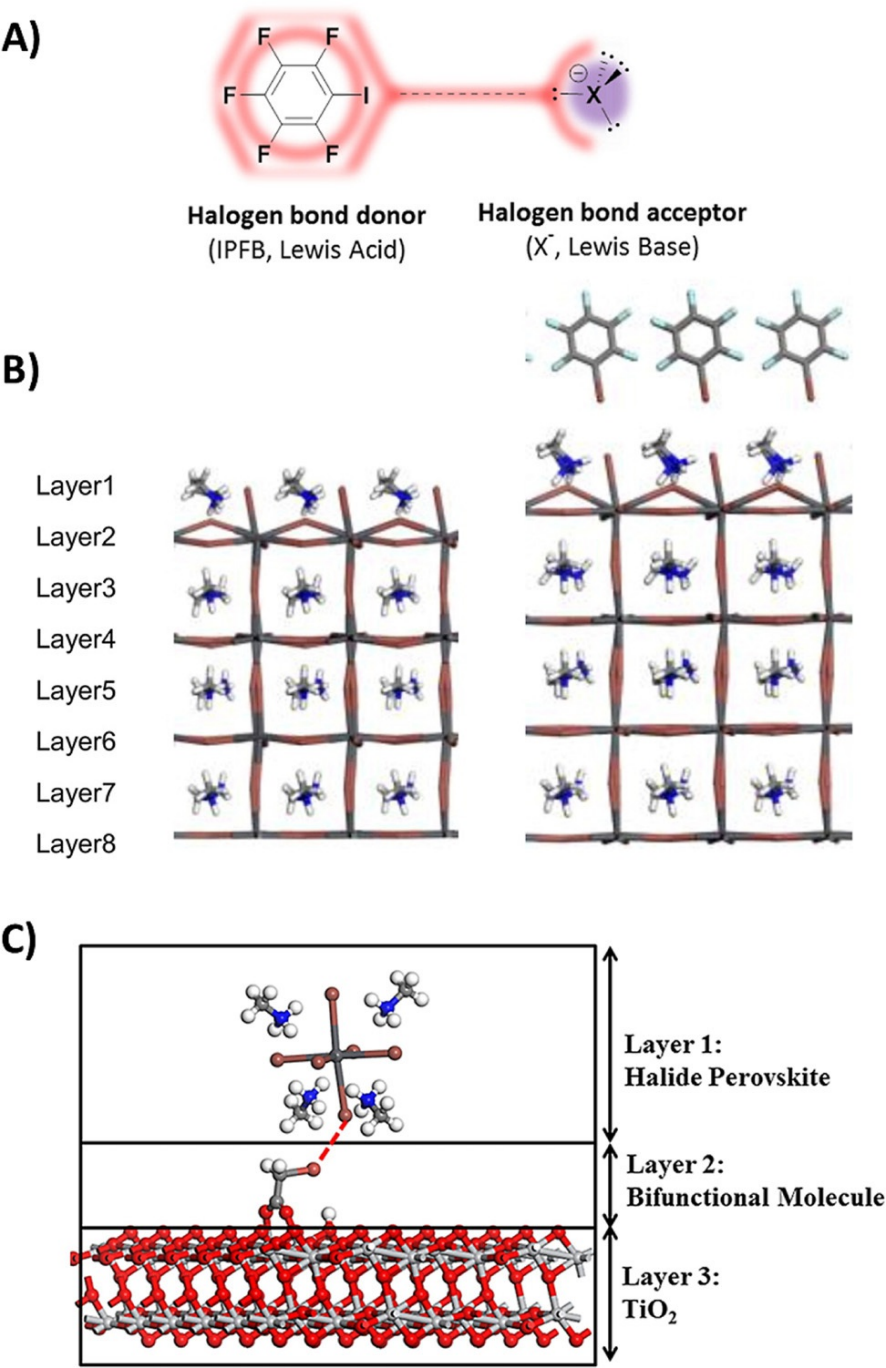
A) Schematic view of the XB between iodopentafluorobenzene (IPFB, XB‐donor) and a generic halide anion (X^−^=I^−^, Br^−^, Cl^−^, XB‐acceptor). Adapted with permission from ref. [40]. B) Optimized geometries of CH_3_NH_3_PbI_3_ perovskite surface with (right) and without (left) IPFB functionalization, and the related effect on the lattice structure. Adapted with permission from ref. [41]. C) The perovskite/bromoacetate/TiO_2_ tri‐layer structure highlights (red dashed line) the XB between the bromoacetate molecule and the halide perovskite layer. Adapted with permission from ref. [42].

Undercoordinated halide anions and migratory species may exist on the surface of perovskite grains. These superficial defects can trap positive charges and holes, promoting non‐radiative charge recombination. XB may provide passivation of these undercoordinated halide anions and, at the same time, improve material crystallinity and enhance the efficiency and stability of the devices. The first example of perovskite surface passivation through XB was reported by Abate et al.^[40]^ back at the dawn of the PSC era. Their work used iodopentafluorobenzene (IPFB) to passivate the under‐coordinated halide ions, which acted as hole traps on the perovskite surface (Figure 2 A). This treatment led to a definite performance improvement in devices. IPFB, indeed, can bind, through XB, the I^−^ ions shielding their electrostatic charge and reducing the accumulated charge at the interface, therefore suppressing hole recombination and promoting charge transfer. These results, later on, motivated Zhang et al.^[41]^ to study the effect of XB passivation in PSCs from a theoretical point of view. Their work confirmed the role of XB in passivating the CH_3_NH_3_PbI_3_ perovskite surface and demonstrated its effectiveness in modulating the crystallinity of perovskite films. Upon adsorption of IPFB, while the inner layers of the perovskite see negligible changes, on the top layer, the Pb‐I‐Pb angle increases and the Pb−I bond lengths are reduced, as shown in Figure 2 B. In this way, the PbI_6_ surface octahedra, which generally adopt a distorted geometry in the bare perovskite, upon interaction with IPFB, become less distorted and more similar to the bulk structure.

### 3.2 XB‐Donors as Organic Interfacial Modifiers

Recently, the same group investigated the effects of haloacetate molecules (chloro‐, bromo‐, and iodoacetates) as bifunctional interfacial modifiers between TiO_2_ and perovskite layers.^[42]^ Haloacetates can act as bifunctional ligands, forming XBs with the halides of the perovskite through the halogen atom and covalent bonds with the TiO_2_ substrate through the carboxylate group (Figure 2 C).

This interaction enhances the interfacial contact between the two materials, thus reducing lattice mismatch and improving the interfacial charge transfer properties. Similar results were also found by Dai et al.,^[43]^ who inserted a bifunctional interfacial modifier between SnO_2_ and perovskite. Such modifier has Si(OCH_3_)_3_ as anchoring group to the SnO_2_ and an iodine‐terminated alkyl chain for the interaction with the perovskite. Also in this case, the presence of XB improved the interfacial adhesion affording higher efficiency and improved operational stability.

### 3.3 XB‐Donors as Crystallization Modulators

**Figure 3:**
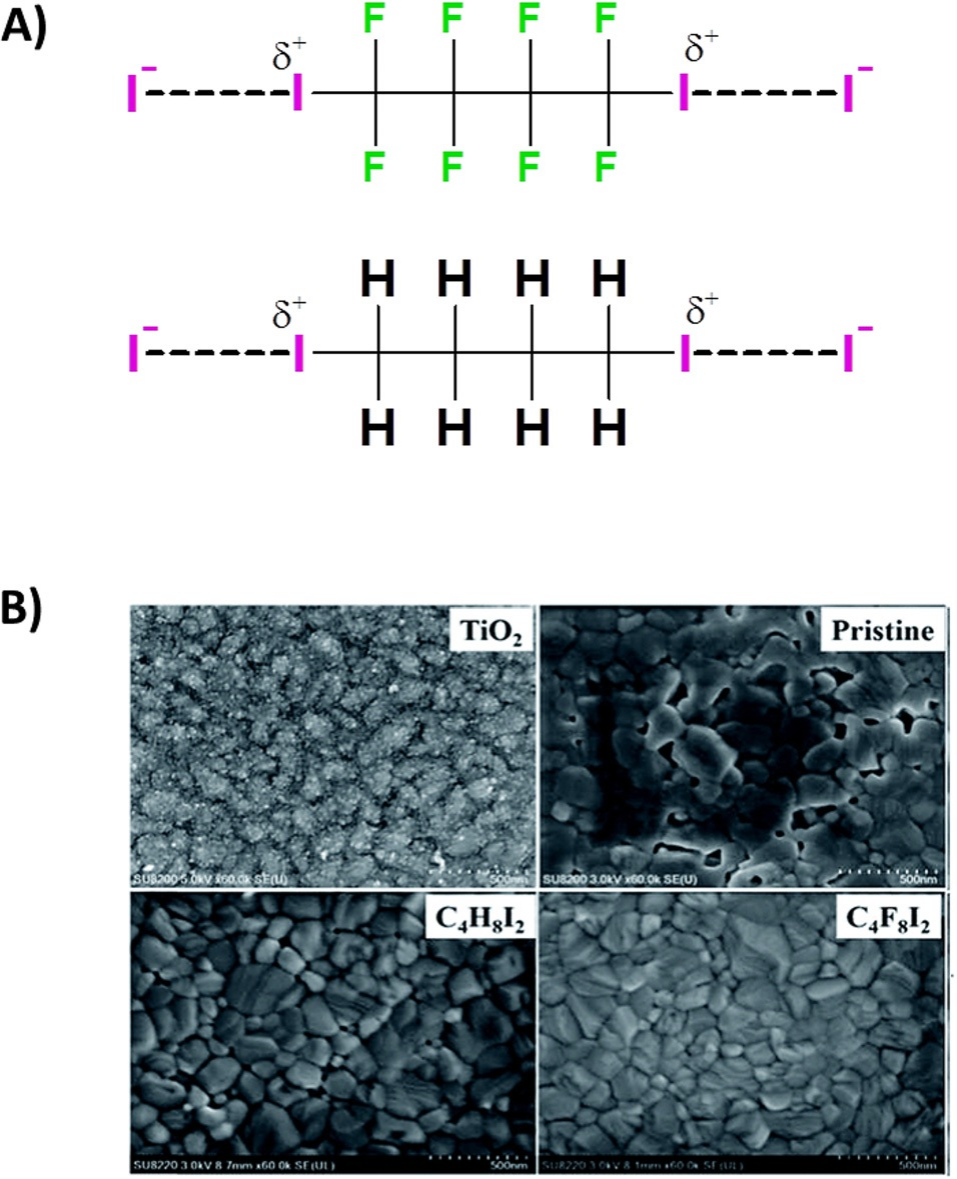
A) Schematic representation of the XB between the additives (C_4_F_8_I_2_, top, and C_4_H_8_I_2_, bottom, XB‐donors) and iodine anions (XB‐acceptors) in the CH_3_NH_3_PbI_3_ precursor solution. B) Top‐view, scanning electron microscopy images of neat TiO_2_ and various CH_3_NH_3_PbI_3_ films after thermal annealing. Adapted with permission from ref. [44].

Experimental evidence of the role of XB‐donors in modulating the crystallization of perovskite films and improving the resulting photovoltaic performances has been provided by Bi et al.^[44]^ They added either diiodoperfluorobutane (I(CF_2_)_4_I) or diiodobutane (I(CH_2_)_4_I) as XB‐donors in the CH_3_NH_3_PbI_3_ perovskite precursor solution (Figure 3 A). Their results revealed that upon XB with these additives, the concentration of free I^−^ anions in the precursor solution is lowered, thus suppressing the formation of iodoplumbate complexes (e.g., PbI_3_^−^, PbI_4_^2−^, and higher‐coordination compounds), which generally hinder the crystallization of perovskites. Improved crystallinities and morphologies were obtained for the perovskite film, which showed a more compact and uniform surface (Figure 3 B). This is reflected in longer photoluminescence (PL) decay and a higher steady‐state PL peak, suggesting further suppression of the surface traps. Space‐charge‐limited current measurements indicated better charge transport and suppressed recombination. Moreover, a substantial reduction of hysteresis was noticed, which agrees with limited ion mobility under electric field due to suppressed non‐stoichiometry and surface charge trapping. Finally, the improvements mentioned above resulted in enhanced environmental stability for the devices prepared with XB additives.

**Figure 4:**
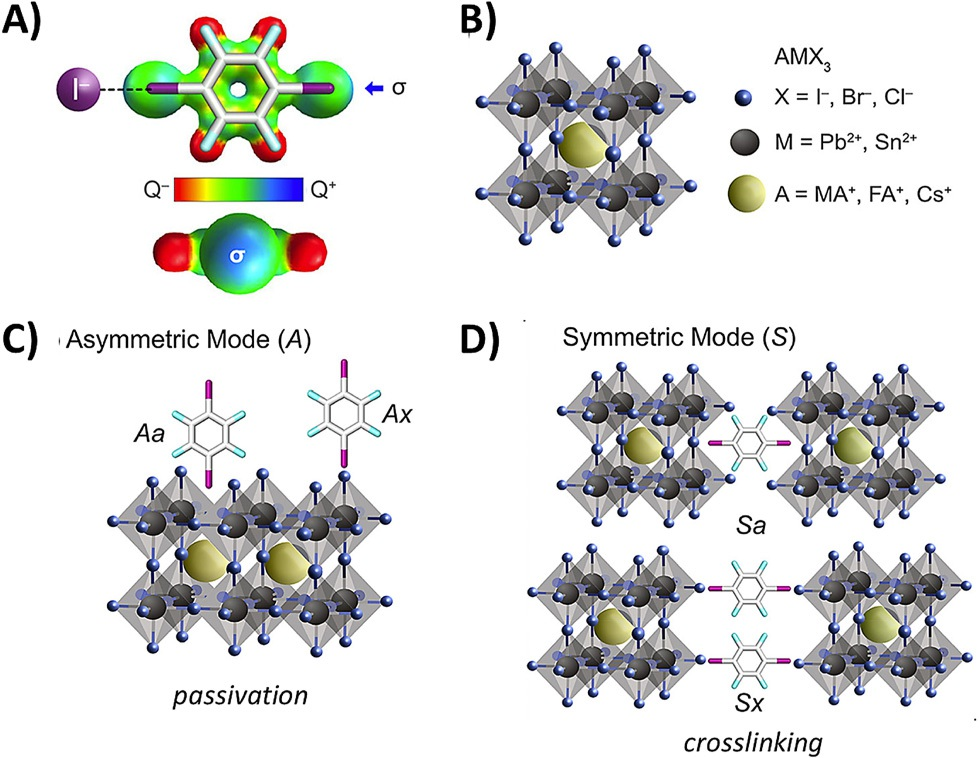
A) Structure and optimized geometry of TFDIB, with the corresponding top and side views of the electrostatic potential surface highlighting the σ‐hole. B) Structure of hybrid perovskites AMX_3_. C, D) Schematic representations of different binding modes of TFDIB to the perovskite, i.e., C) asymmetric mode (A) and D) symmetric crosslinking mode of the phases (S). Adapted with permission from ref. [45].

The general applicability of this concept was further demonstrated by Ruiz‐Preciado et al.^[45]^ by introducing 1,2,4,5‐tetrafluoro‐3,6‐diiodobenzene (TFDIB) as bifunctional supramolecular modulator (Figure 4). TFDIB was chosen for its hydrophobicity and capability to strongly bind perovskite halides through XB. If added to the precursor solution or used as an interlayer between perovskite and charge transport materials, it resulted in improved *V*_oc_ and enhanced long‐term operational stability, with 80 % efficiency retained after 500 h of continuous illumination. The improved stability was ascribed to the hydrophobic nature of the molecule and to its effect on the stabilization of the FAPbI_3_ α‐phase (FA=formamidinium), as shown by X‐ray diffraction measurements. This work also provided a fascinating insight into the atomic‐level mode of interaction of TFDIB and perovskite, thanks to detailed NMR analyses and DFT calculations, which evidenced the role of crosslinking XB. Their results showed that the modulator preferably coordinates PbI_2_ or MAI (MA=methylammonium) through both symmetric and asymmetric coordination binding modes. At the same time, there was no evidence of the formation of the FAI–TFDIB complex. A similar crosslinking mechanism has also been proposed for 2‐bromo‐6‐fluoronaphthalene introduced as an interfacial modulator for printable hole‐conductor‐free mesoscopic PSCs through post‐treatment.^[46]^

### 3.4 XB Self‐Assembled Monolayers

Molecules capable of coordinating though XB are also excellent candidates to form self‐assembling monolayers (SAMs), which are of particular interest because of their stability and ordered distribution. An example of the advantages following the employment of XB‐driven SAMs on perovskites is given by the work of Wolff et al.,^[47]^ where iodoperfluoroalkanes of different chain lengths (IPFC_*n*_ with *n*=8, 10, 12) were used to functionalize the surface of perovskites in inverted PSCs. The results showed a significant *V*_oc_ improvement, which the authors ascribed to reducing non‐radiative recombination and gain in charge separation by looking at absolute PL, time resolved‐PL, and surface photovoltage measurements. Moreover, increased stability was also reported for the treated devices that were able to withstand harsh stress conditions like 250 h of continuous illumination at 85 °C, and retain 95 % efficiency after 4 months of storage in ambient conditions.

IPFC_10_ has also been employed by Canil et al. in a more recent work showing that it is possible to exploit SAMs to tune the perovskite energy levels continuously. The authors demonstrated that it was possible to control the deposition kinetics by managing the molecules' deposition parameters, i.e., solution concentration or dipping time. Thus the magnitude and direction of the energy levels shift. XB allowed obtaining ordered and stable monolayers, proving very useful for this kind of application. It enhances the ability to control the deposition and fine‐tuning the perovskite energy level alignment.^[48]^

This concept was later on further developed by the same group, who obtained enhanced stability and performance of the devices by directly incorporating in the hole transport layer (HTL) XB‐donor groups able to interact with the perovskite.^[49]^ Thanks to the presence of XB, the HTL can form a more ordered and compact layer, resulting in a stronger interfacial dipole, reduced energetic offset for hole transport, and suppression of recombination processes. The improved interface also increased the resilience against moisture and solvent, affording outstanding operational stability with a projected lifetime more than two‐fold the standard devices.^[50]^

### 3.5 2D‐HOIHPs

**Figure 5:**
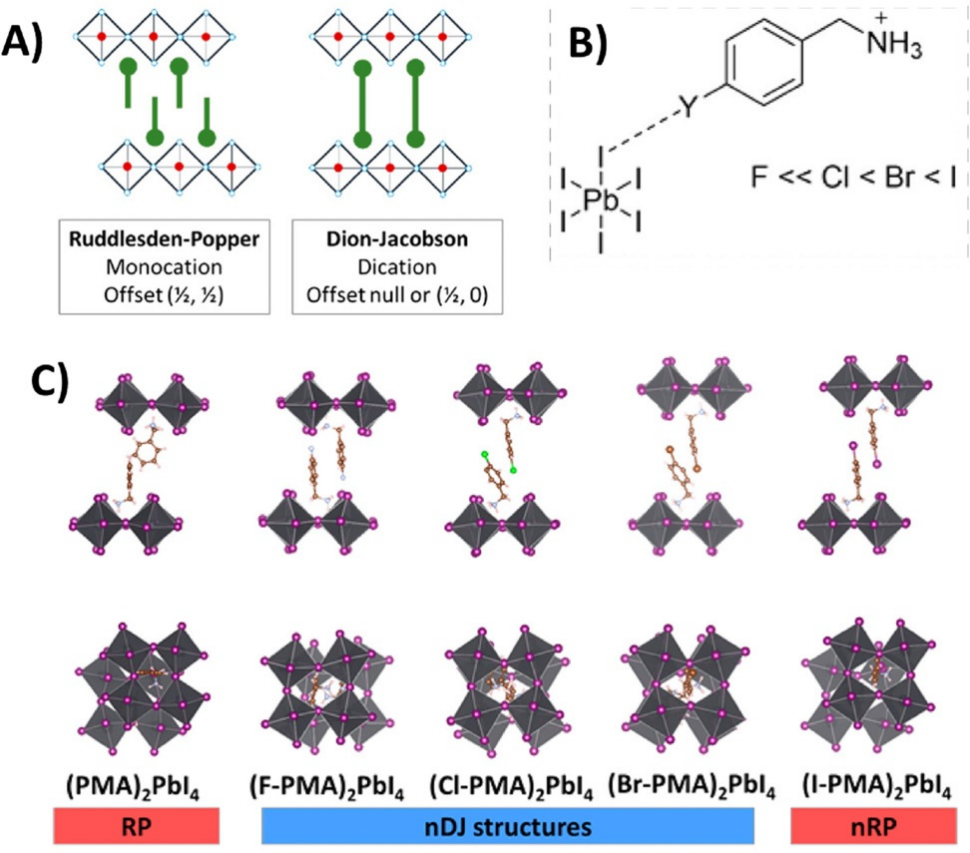
A) Schematic representations of Ruddlesden–Popper (RP) and Dion–Jacobson (DJ) 2D‐HOIHP. B) Schematic representation of the XB between an iodide ion of the inorganic sheet and the halogenated benzylammonium cation 4‐Y‐C_6_H_4_CH_2_NH_3_ (Y‐BzA, Y=F, Cl, Br, I). C) Crystal structures of (Y‐BzA)_2_PbI_4_ showing the near‐DJ (nDJ) and near‐RP (nRP) arrangements obtained with halogenated BzAs. Adapted with permission from ref. [51].

Finally, XB has also been exploited to modulate the crystal structure of 2D‐HOIHPs, which consist of single perovskite sheets separated by organic cations.^[51]^ According to their crystal structure, 2D‐HOIHPs can be classified as either Dion–Jacobson (DJ) or Ruddlesden–Popper (RP) structures (Figure 5 A). RP‐HOIHPs feature monovalent organic cations interdigitating between adjacent inorganic layers of MX_6_ octahedra with a staggered arrangement. Differently, the DJ‐HOIHPs feature divalent organic cations between the layers, which adopt an eclipsed arrangement. Tremblay et al.^[51]^ reported the crystal structures of a family of 2D hybrid structures (4‐Y‐C_6_H_4_CH_2_NH_3_)_2_PbI_4_ (where Y=F, Cl, Br, I), and demonstrated that despite the use of a monovalent organic cation, for Y=F, Cl, and Br the resulting 2D perovskites gave a DJ structure. Conversely, when iodine was used, i.e., Y=I, for which stronger XB is expected, an RP‐like structure was obtained.

This study suggests that tuning the interaction strength between organic and inorganic layers allows controlling the perovskite stacking pattern in HOIHPs. A similar approach has been adopted by Fu et al.,^[52]^ who recently used a monovalent tetrafluoro iodoaromatic synthon to increase the XB‐donor ability of the cation in a 2D/3D hybrid perovskite. The interfacial XB between 2D and 3D perovskites anchors the iodide anions at the grain boundaries, which suppresses phase separation and significantly improves the long‐term operational stability of the photovoltaic devices.

## 4 Conclusion

As is often the case for rapidly emerging and growing fields, many researchers from different backgrounds join in, attracted by the rapid pace of citations growth. While we recognize that some multidisciplinary areas, such as PSCs, may benefit from inputs from different backgrounds, it is our opinion it has always to be done in keeping with the established understanding of phenomena and correct use of related terminology. Our critical review of current literature about the use of XB in HOIHPs has revealed that phenomena misinterpretation and term misuses may occur.

As an example, Tang et al. reported that N⋅⋅⋅Br halogen bonds, between N‐doped‐C heterostructures and the Br^−^ atoms from CsPbBr_3_ (CPB), chemically immobilize CPB on N–C layers, enhancing the aqueous stability of CPB@Co_3_O_4_/N‐C by prohibiting CPB decomposition.^[53]^ In our opinion, it is more likely that this interaction can be ascribed to a genuine hydrogen bond, where the nucleophilic Br^−^ ion is behaving as the electron donor towards the pyrrolic H atom. Paek et al., instead, reported that fluoro‐substituted 2D materials bearing perfluoroaromatic synthons are more effective than monofluorinated ones, resulting in strong interaction with the 3D perovskite, which induces a highly in‐plane oriented growth of the crystals while preserving excellent hole transfer.^[54]^ We found these results extremely interesting. However, they may be more likely related to the peculiar electron distribution in perfluorophenyl rings, which usually behave as electron‐acceptors in anion⋅⋅⋅π interactions.^[55]^ Similar interactions were also wrongly attributed to XB when using 2,3,5,6‐tetrafluoro‐7,7,8,8‐tetracyano‐quinodimethane (F4TCNQ) as interlayer between the HTL and perovskite active layer in PSCs.[^56^, ^57^]

In summary, we have highlighted the potential that XB has to bring great and reproducible advantages for developing highly stable PSCs. Common features among most of the reported examples are improved crystallization and stability, reduction of the surface trap states, and the possibility of forming ordered structures and layers. These results are of utmost interest considering the variety of perovskite compositions and device structures and the sensitivity of the current materials to external factors. Nevertheless, there is still much research needed in both XB and HOIHPs. In particular, an atomic/molecular understanding is highly needed for fully exploiting the advantages of the combination of these two rapidly growing research fields. However, the future of XB in HOIHPs looks bright.

## Conflict of interest

The authors declare no conflict of interest.

## Biographical Information

*Laura Canil is Ph.D. candidate at the department of “Novel materials and interfaces for photovoltaic solar cells” at the Helmholtz‐Zentrum Berlin in Germany. She obtained her master's degree in physics in 2017 from the University of Padova, Italy, with a thesis on electrochemical growth of silicon nanostructures performed at the institute AMOLF in Amsterdam, Netherlands. Now her research focuses on characterization and tuning of the interfacial properties in perovskite solar cells*.

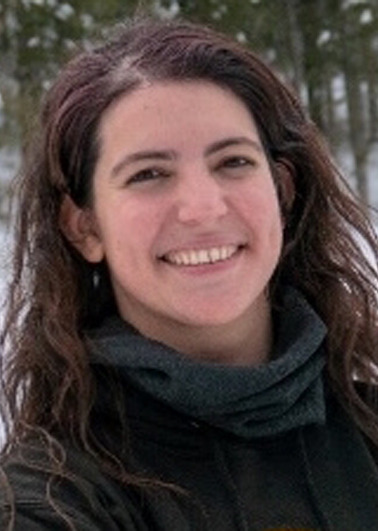

## Biographical Information

*Antonio Abate is director of the department of “Novel Materials and Interfaces for Photovoltaic Solar Cells” at the Helmholtz‐Centrum Berlin in Germany, Professor at the University of Naples Federico II in Italy, and Visiting Professor at Fuzhou University in China. His research focuses on halide perovskite solar cells. Before his current position, he led the solar cell research at the University of Fribourg in Switzerland. He was a Marie Skłodowska‐Curie Fellow at École Polytechnique Fédérale de Lausanne in the group of Prof. Michael Grätzel and postdoctoral researcher at the University of Oxford and the University of Cambridge*.

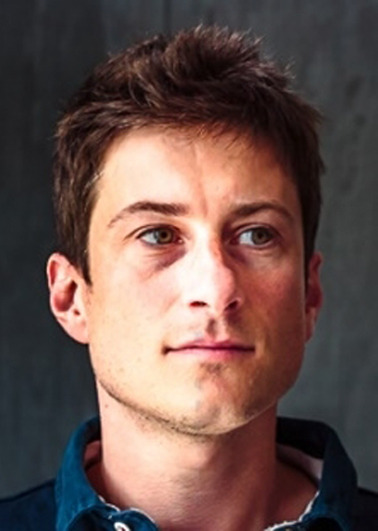

## Biographical Information

*Giancarlo Terraneo gained his PhD in Chemistry at the Department of Chemistry—Università degli Studi di Milano in 2006 under the supervision of Prof. Anna Bernardi. He is full professor at the Department of Chemistry, Materials, and Chemical Engineering “Giulio Natta”—Politecnico di Milano. His research interests are in crystal engineering, supramolecular chemistry, and crystallography*.

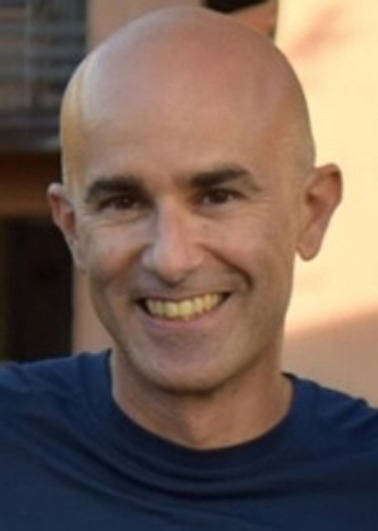

## Biographical Information

*Gabriella Cavallo is Associate Professor at Politecnico di Milano since 2018. She obtained her PhD in Chemistry in 2006. After two years of activity in Pirelli Labs, a research unit of Pirelli S.p.A., she became post‐doctoral researcher at Politecnico di Milano in 2008. She has been Team Leader at the Center of Nano Science and Technology of the Italian Institute of Technology and visiting researcher at the VTT—Technical Research Centre of Finland. Her research activity is focused on the study of structure–property relationships of new fluorinated functional materials based on non‐covalent interactions*.

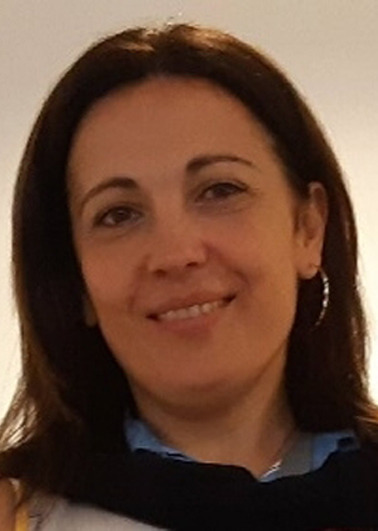

## Biographical Information

*Pierangelo Metrangolo is full professor at Politecnico di Milano since 2011, where he is Executive Deputy Head of the Department of Chemistry, Materials, and Chemical Engineering “Giulio Natta”. He also holds a position as visiting professor at the Centre of Excellence in Molecular Engineering of Biosynthetic Hybrid Materials of Aalto University, Finland, and at the VTT‐Technical Research Centre of Finland. Since 2013 he is European Research Council grantee with the projects “FOLDHALO—Folding with Halogen Bonding”, and “MINIRES—A Minimalist Elastomeric Peptide” aimed at investigating the relevance of biomolecular halogen bonds*.